# Evaluation of the Relationship Between Orexin A, Peptide YY, AgRP, and POMC Levels and Sleep Disorders in Children with Malnutrition

**DOI:** 10.3390/nu18030377

**Published:** 2026-01-23

**Authors:** Anna Carina Ergani, Mustafa Esad Tezcan, Ümmügülsüm Can, Emine Arslan Kılıçoğlu

**Affiliations:** 1 Department of Pediatric Gastroenterology, Konya City Hospital, 42020 Konya, Turkey; 2 Department of Child and Adolescent Mental Health, Faculty of Medicine, Selcuk University, 42250 Konya, Turkey; esadaxe@gmail.com; 3 Department of Clinical Chemistry, Konya City Hospital, 42020 Konya, Turkey; cangulsum@yahoo.com; 4 Child Development Program, Department of Child Vocational, School of Health Services, KTO Karatay University, 42020 Konya, Turkey; emine.arslan@karatay.edu.tr

**Keywords:** appetite-regulating peptides, Children’s Eating Behaviour Questionnaire (CEBQ), Children’s Sleep Habits Questionnaire (CSHQ), malnutrition, orexin A, peptide YY (PYY)

## Abstract

Background: Malnutrition and sleep disturbances are common in childhood and are associated with neuropeptides that regulate appetite and circadian rhythms. Hypothalamic peptides such as orexin A, agouti-related protein (AgRP), proopiomelanocortin (POMC), and peptide YY (PYY) play important roles in energy balance and eating behavior; however, their specific functions in pediatric malnutrition remain unclear. This study aimed to determine the levels of these peptides in malnourished children and to examine their relationship with eating and sleep behaviors. Methods: This case–control, cross-sectional study included 99 children aged 5–15 years diagnosed with malnutrition and 85 age-matched healthy controls. Blood samples were collected from all participants, and peptide levels were measured using ELISA. Additionally, the Children’s Eating Behaviour Questionnaire (CEBQ) and the Children’s Sleep Habits Questionnaire (CSHQ) were administered to assess eating patterns and sleep behaviors. Statistical comparisons and hierarchical logistic regression analyses were performed. Results: Orexin A and PYY levels were significantly higher in malnourished children than in controls (*p* < 0.001). No significant group differences were found for AgRP and POMC, although AgRP tended to be lower and POMC higher in the malnutrition group. Regression analyses identified orexin A and PYY as significant predictors of malnutrition. Orexin A showed a positive correlation with emotional undereating. Sleep habits did not differ significantly between groups. Conclusions: Elevated orexin A and PYY levels may function as potential neuropeptidergic biomarkers of malnutrition. Their association with emotional eating highlights the psychobiological components of malnutrition. Further longitudinal studies are warranted to clarify causal mechanisms and support clinical translation.

## 1. Introduction

Malnutrition exists in a bidirectional interaction with the sleep–wake cycle and influences both nutritional status and sleep quality through the regulation of peptides, such as orexin A, leptin, and ghrelin. This dynamic creates significant and lasting effects on health throughout the life course, from childhood to adulthood [1,2]. Malnutrition is defined as a nutritional disorder that leads to measurable adverse outcomes in body mass and functions due to a deficiency or excess of protein, energy, or other nutrients [3,4].

Sleep is a cyclical and active physiological process that regulates many vital functions, including growth, learning, memory, academic performance, immune system function, and the clearance of neurotoxic substances [5]. The normal sleep–wake cycle (circadian rhythm) is regulated by the suprachiasmatic nucleus in the hypothalamus, various neurotransmitters (i.e., acetylcholine, histamine, dopamine, glutamate, norepinephrine, serotonin, and GABA), and melatonin in response to light-dark changes [6]. Although the mechanisms by which sleep regulates the effects on appetite and hunger hormones are not yet fully understood, the current findings suggest that sleep may alter food intake by influencing the excitability of appetite neurons and the levels of hunger hormones [7,8].

Sleep is a fundamental physiological process for the growth and development of children. Sleep disorders in childhood have been reported to be associated with both malnutrition and obesity [7]. However, it remains unclear whether sleep disorders lead to disordered eating behaviors or whether eating disorders disrupt sleep patterns. Regardless of the presence of malnutrition, one in four children may experience sleep disturbance at some point in their lives [9].

Eating behavior is regulated by a system centered in the hypothalamus that determines food intake in response to the body’s internal energy status [10]. The hypothalamus contains various neural centers, including the lateral hypothalamic nucleus, which functions as the “hunger center,” and the ventromedial nucleus, known as the “satiety center.” Additionally, the paraventricular nucleus and the hypothalamic arcuate nucleus (ARC) are structures where numerous hormones secreted from the gut and adipose tissue converge to regulate food intake and energy expenditure.

Within the ARC are two distinct groups of neurons that regulate appetite: anorexigenic (appetite suppressing) POMC neurons and orexigenic (appetite stimulating) neuropeptide Y (NPY)/AgRP neurons [11]. The ARC plays a crucial role in appetite regulation by integrating inputs from the vagus nerve and various circulating molecules, such as orexin, ghrelin, leptin, and insulin. The activation of the AgRP neurons increases appetite and slows metabolism [12]. At the same time, AgRP competitively inhibits the melanocortin receptors, particularly MC3-R and MC4-R, by suppressing the appetite-suppressing effects of POMC. Conversely, POMC enhances satiety and increases energy expenditure by promoting the release of melanocyte-stimulating hormone (MSH) [13]. Finally, PYY is an anorexigenic peptide that is synthesized both in the gastrointestinal system and the central nervous system. It contributes to appetite suppression through its effects on the ARC [14].

Orexin, leptin, and ghrelin are key hypothalamic peptides that link appetite regulation with the sleep–wake cycle and energy homeostasis [15]. Among these, orexin A plays a central role in maintaining wakefulness and regulating energy balance and feeding behavior, and its dysfunction has been associated with various pathological conditions, including narcolepsy [9,16]. However, the relationship between orexin dysfunction and malnutrition has not yet been clearly established.

In contrast, PYY and POMC exert anorexigenic effects by promoting satiety. While sleep disorders may acutely increase the secretion of orexigenic peptides, chronic sleep disturbances reduce hypothalamic receptor sensitivity and disrupt appetite regulation [17]. Additionally, sleep deprivation suppresses POMC activity by reducing the leptin and melatonin levels [18]. Malnutrition exacerbates peptide system desensitization due to energy deficiency, thereby leading to inadequate physiological responses. This disruption of the appetite signaling mechanism can cause significant disturbances in both the nutrition and sleep rhythms [19]. Therefore, monitoring the levels of orexin A, peptide YY (PYY), agouti-related protein (AgRP), and proopiomelanocortin (POMC) in individuals with malnutrition and sleep disturbances is of clinical importance for understanding underlying pathophysiological mechanisms. These peptides were selected because they collectively represent the principal neuropeptidergic pathways involved in appetite regulation, energy homeostasis, and the sleep–wake cycle, encompassing both central hypothalamic and peripheral gut-derived signals.

A review of the current literature revealed that the number of studies that evaluated the levels of appetite- and sleep-regulating peptides in malnourished children is limited. This study aimed to fill this research gap by comparing the plasma levels of peptides such as orexin A, POMC, AgRP, and PYY in children with malnutrition to those of healthy controls and by investigating the relationships between these peptide levels and eating and sleep behaviors in children.

## 2. Materials and Methods

### 2.1. Participants and Procedures

**Figure 1:**
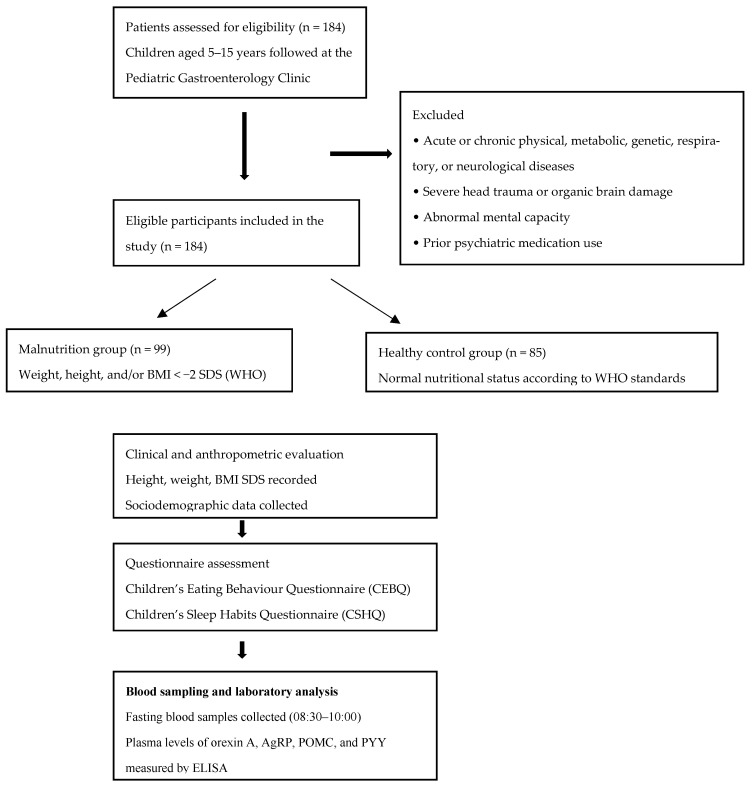
Patient inclusion flowchart (n = number).

Patients aged 5–15 years who were followed at a pediatric gastroenterology clinic were included in the study based on the following criteria. The exclusion criteria comprised the presence of acute or chronic physical, metabolic, genetic, respiratory, or neurological diseases (e.g., diabetes mellitus, hypertension, epilepsy, and cerebral palsy), severe head trauma or organic brain damage, clinically abnormal mental capacity, and prior use of any psychiatric medications. Considering these criteria, male and female patients with weight, height, and/or a body mass index (BMI) below −2 standard deviations (SDS) according to the World Health Organization (WHO) growth standards were included in the malnutrition group [4]. The control group included children aged 5–15 years without a diagnosis of chronic disease who presented with complaints other than malnutrition (acute abdominal pain or acute diarrhea) or were referred from external centers with suspected growth retardation but had age-appropriate anthropometric measurements. Nutritional status was classified as normal according to World Health Organization (WHO) criteria. Participant selection is summarized in Figure 1.

The same exclusion criteria mentioned above were applied to the control group. The study procedures were thoroughly explained to the parents and participants, and written informed consent was obtained from those who agreed to participate. A total of 99 malnourished patients (aged 5–15 years) and 85 healthy controls (aged 5–15 years) were included in the study. The entire study process was conducted in accordance with the Helsinki Declaration. This study was approved by an independent medical ethics committee (date: 7 March 2024; approval no: 2024/012) and was conducted in the Pediatric Gastroenterology Department of Konya City Hospital between April 2024 and April 2025 as part of routine clinical follow-up.

### 2.2. Diagnosis and Symptom Evaluation

To evaluate the eating behaviors of the children, the Children’s Eating Behavior Questionnaire (CEBQ) was administered to the parents in both cohorts. The CEBQ comprises 35 Likert-scale items that respondents rate on a 5-point scale (e.g., 1 = never, 5 = always). The 35 items are systematically categorized into eight distinct subscales: (1) food responsiveness (5 items), (2) enjoyment of food (4 items), (3) slowness in eating (4 items), (4) food fussiness (6 items), (5) satiety responsiveness (5 items), (6) emotional overeating (4 items), (7) emotional undereating (4 items), and (8) desire to drink (3 items) [20]. Extremes in scoring on these subscales are indicative of maladaptive eating behaviors. Notably, the instrument lacks validated cutoff thresholds for the classification of dysfunctional patterns. The psychometric properties of the CEBQ have been substantiated in the Turkish population by Yilmaz et al., who reported Cronbach’s alpha coefficients ranging from 0.61 to 0.84, thus confirming acceptable reliability and validity [21].

To assess the sleep habits of the children, the Children’s Sleep Habits Questionnaire (CSHQ) was administered to the parents in both groups. Developed by Owens et al. in 2007 the shortened version of this instrument consists of 33 items designed to evaluate sleep habits and related problems [8]. The scale includes eight subscales: bedtime resistance, sleep onset delay, sleep duration, sleep anxiety, night wakings, parasomnias, sleep-disordered breathing, and daytime sleepiness. The questionnaire is completed retrospectively by parents, who rate their child’s sleep behaviors over the prior week. Items are generally scored as follows: “usually” (5–7 times per week) = 3, “sometimes” (2–4 times per week) = 2, and “rarely” (0–1 time per week) = 1. However, items 1, 2, 3, 10, 11, and 26 are reverse scored, and items 32 and 33 are coded differently with “does not fall asleep” = 0, “very sleepy” = 1, and “falls asleep suddenly” = 2. A total score of 41 or above is considered clinically significant for sleep disturbances. Additionally, the questionnaire contains three open-ended questions regarding the child’s bedtime, total sleep duration, and duration of wakefulness during the night.

The instrument also assesses behavioral and emotional problems related to hyperactivity, inattention, irritability, oppositional behavior, restlessness, mood disturbances, anxiety, and somatic complaints through nine items rated by parents on a frequency scale of “rarely”, “sometimes”, and “often”. The reliability and validity of the Turkish version of the CSHQ were established by Perdahli Fis et al., who reported Cronbach’s alpha coefficients ranging between 0.68 and 0.80, thus indicating satisfactory internal consistency [22].

Following the assessment, sociodemographic data forms were completed by the clinician for both groups. Anthropometric measurements were performed for all of the children to calculate their height, weight, and BMI standard-deviation scores (SDS). Routine blood samples were collected to analyze for malnutrition-related parameters, including albumin, hemoglobin (Hgb), vitamin D, folic acid, and vitamin B12. Additionally, plasma samples were obtained for the peptides to be analyzed in the study, namely AgRP, POMC, PYY, and orexin A. The evaluation of participants from both groups was conducted by a specialist pediatrician.

### 2.3. Collection of Blood Samples

Blood samples from the participants were collected between 8:30 and 10:00 a.m. following an eight-hour fasting period. The blood samples were then centrifuged at 2000–3000 rpm for 20 min to separate the plasma. The separated plasma samples were stored at −80 °C in Eppendorf tubes until analysis. Human POMC, orexin A, AgRP, and PYY enzyme-linked immunosorbent assay (ELISA) kits (Shanghai Korain Biotech Co., Ltd., Shanghai, China) were used to measure the plasma peptide levels. The levels of these peptides in the participants blood samples were determined using the ELISA method in an accredited medical biochemistry research laboratory.

### 2.4. Statistics

The Python programming language (version 3.14.1, Python Software Foundation, Wilmington, DE, USA, https://www.python.org/) was employed for the hierarchical logistic regression analysis, while statistical analyses were performed using SPSS version 29.0 (SPSS Inc., Chicago, IL, USA). Clinical, demographic, and hematological variables were compared between the groups based on their distributions, as assessed by the Student’s *t*-test or the Mann–Whitney U test. The chi-square test was applied for the categorical variables. Normality was evaluated using skewness and kurtosis values within the range of −2 to +2 [23].

Hierarchical logistic regression models were constructed utilizing the maximum likelihood estimation method. Model explanatory power and goodness-of-fit were assessed using pseudo R^2^ and log-likelihood ratio (LLR) tests. The independent variables were first normalized prior to inclusion in the models. The analysis results are presented as model coefficients (β), standard errors (SE), Wald Z statistics, *p*-values, and 95% confidence intervals (CI). Age, sex, and BMI were incorporated as covariates in the hierarchical regression models. Statistical significance was set at *p* < 0.05 with a 95% confidence level.

Effect sizes were estimated using Cohen’s d for parametric and nonparametric comparisons and Cramer’s V for categorical variables. According to Cohen’s criteria, effect sizes were interpreted as large (≥0.8), medium (0.5–0.7), small (0.2–0.4), and negligible (<0.2) [24]. Correlations between biochemical parameters and clinical variables were also assessed. Pearson’s correlation coefficient was used for the parametric data, while Spearman’s rank correlation coefficient was applied for the nonparametric data to evaluate the relationships between variables.

## 3. Results

A total of 184 individuals were included in the study, comprising 85 healthy controls and 99 malnourished children. There were no statistically significant differences between the malnourished patient and healthy control groups in terms of socioeconomic status, age, sex, paternal and maternal age, or educational levels. However, significant differences were observed between the two groups in terms of weight, height, and BMI standard deviation scores (weight SDS, *p* < 0.001; height SDS, *p* < 0.001; BMI SDS, *p* < 0.001). The percentile and standard deviation values for the weight, height, sex, age, and BMI of both groups are presented in Table 1.

Children who score 42 and above on the CSHQ assessment had sleep problems. Accordingly, no statistical difference was found between the malnourished patient group and the healthy control group. However, the total CSHQ scores for both the malnourished patients and the healthy control group were above 42 (control group = 54.41 ± 6.60, malnourished children = 55.24 ± 6.97), which indicated that both groups had sleep disturbances. There was no statistically significant difference between the two groups in terms of bedtime resistance, sleep duration, sleep anxiety, night awakenings, parasomnias, sleep-disordered breathing, or daytime sleepiness (Table 2).

The comparison of the subscales of the CEBQ revealed no statistically significant differences between the malnourished patient group and the control group in terms of food responsiveness, emotional overeating, desire to drink, enjoyment of food, or food fussiness scores (Table 2). The plasma albumin and vitamin D levels were found to be significantly lower in the malnourished patient group compared to the healthy control group (*p* < 0.001). No statistically significant differences were observed between the malnourished patient group and the healthy control group in terms of plasma hemoglobin, vitamin B12, or folic acid levels.

**Figure 2:**
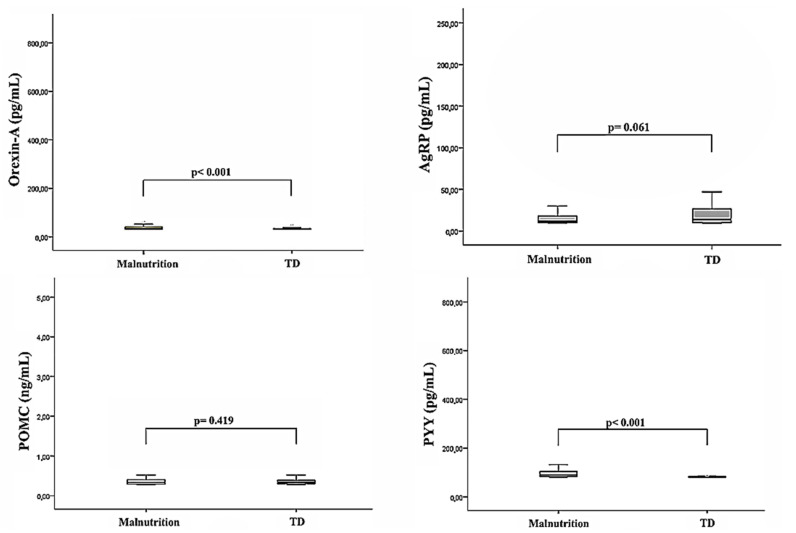
Box plots showing the distribution of the orexin A, proopiomelanocortin (POMC), agouti-related protein (AgRP), and peptide yy (PYY) levels in the malnourished children and typically developing (TD) healthy controls. The Mann–Whitney U test was used to compare the peptide levels between the two groups.

The plasma orexin A and PYY levels were found to be significantly higher in the malnourished patient group compared to the healthy control group (*p* < 0.001). In contrast, no statistically significant differences were observed between the two groups in terms of AgRP and POMC levels. However, in patients with malnutrition, a decrease in AgRP levels and an increase in POMC levels were noted. The plasma levels of orexin A, PYY, AgRP, and POMC in the malnourished patients and healthy controls are shown in Table 3 and Figure 2.

The correlation between the CEBQ and CSHQ scores and the POMC, orexin A, AgRP, and PYY levels in the malnourished patients was evaluated using Spearman’s correlation test. Orexin A was found to have a positive correlation with the emotional undereating scores. No correlations were observed among the other variables (Table 4 and Table 5).

In this study, a three-step hierarchical logistic regression analysis was performed to evaluate the levels of appetite-related molecules in malnutrition patients and TD controls. Age, sex, and BMI were included as covariates in the model. In the first step, only the appetite-stimulating molecules orexin A and AgRP were included in the model. In this model, no statistically significant relationship was found between the dependent variable, malnutrition status, and orexin A (β = 0.0041, *p* = 0.286) or AgRP (β = −0.0031, *p* = 0.645) (Table 6).

The pseudo R^2^ value for the overall model fit was calculated as 0.289 (LLR *p* < 0.001). In the second step, the appetite-suppressing molecules POMC and PYY were added as independent variables. POMC (β = −6.3663, *p* = 0.014) and PYY (β = 0.1547, *p* < 0.001) were found to be significant, which indicated that these variables play important roles in determining malnutrition status. The pseudo R^2^ value of the model was calculated as 0.433 (LLR *p* < 0.001).

In the final step, all molecules (orexin A, AgRP, POMC, and PYY) were included in the model. In this model, POMC (β = −6.2495, *p* = 0.015) and PYY (β = 0.1660, *p* < 0.001) remained significant independent variables. However, orexin A (β = 0.0071, *p* = 0.226) and AgRP (β = −0.0076, *p* = 0.289) were not found to be significant. The pseudo R^2^ value of the model was calculated as 0.448 (LLR *p* < 0.001) (Table 4).

## 4. Discussion

To the best of our knowledge, this is the first study to concurrently investigate circulating levels of the sleep- and appetite-regulating peptides—orexin A, AgRP, POMC, and PYY—in malnourished pediatric patients. Our findings demonstrated that levels of orexin A and PYY were significantly higher in the malnourished group compared to the healthy control group. Based on hierarchical regression analysis that adjusted for age, sex, and BMI as covariates, the POMC and PYY levels were found to be potentially associated with the diagnosis of malnutrition. Additionally, the orexin A levels were positively correlated with the emotional undereating scores.

Orexin A secretion is known to increase under conditions of energy deficiency, including malnutrition, and this elevation is generally interpreted as an adaptive response that promotes arousal and food-seeking behavior to compensate for reduced energy availability [25]. In line with this framework, the higher orexin A levels observed in the malnourished group in our study support the notion of a compensatory neuroendocrine adaptation and are consistent with previous reports [26,27]. Orexin neurons also regulate multiple homeostatic functions, including feeding behavior and energy expenditure [28]. Notably, Edwards et al. demonstrated that intraperitoneal administration of orexin A increased food intake [26], suggesting that orexin A may contribute to hunger-driven responses during energy deficit.

Malnutrition is a systemic stress condition that affects not only energy balance and appetite regulation but also sleep–wake mechanisms. Elevated orexin A levels during energy deficiency may activate hypothalamic arousal centers, potentially prolonging sleep onset latency and disrupting sleep continuity [7]. Given its central role in maintaining wakefulness, orexin A has been suggested to contribute to reductions in both sleep duration and sleep quality. Accordingly, alterations in sleep initiation, total sleep time, sleep continuity, and daytime functioning may be expected to be reflected in higher CSHQ scores [29,30]. However, in the present study, CSHQ scores were similar between malnourished children and healthy controls, suggesting that elevated orexin A levels may have a limited direct effect on subjective sleep quality as assessed by parental reports. Notably, both groups exhibited CSHQ scores above the clinical threshold of 40, indicating the presence of sleep disturbances across cohorts. In addition, the mean sleep duration in the malnourished group was below age-appropriate recommendations [31]. These findings highlight the need for future studies employing objective sleep measures and longitudinal designs to better elucidate the role of sleep duration and quality in energy balance and appetite regulation in malnourished children.

Orexin A is a neuropeptide that influences not only physiological hunger but also emotional and behavioral aspects of eating. Previous CEBQ-based studies have demonstrated an association between elevated orexin A levels and higher emotional undereating scores, a behavior characterized by reduced food intake in response to emotional distress [32]. Although orexin A is generally considered appetite-stimulating, its relationship with emotional undereating may paradoxically contribute to reduced energy intake and the development or persistence of malnutrition during childhood. Nevertheless, given the cross-sectional nature of the available evidence, the causal direction of this association remains unclear and warrants further investigation in longitudinal studies.

In this study, the plasma levels of AgRP, the only orexigenic peptide evaluated, did not differ significantly between malnourished children and healthy controls. Although AgRP is known to promote food intake and energy acquisition [33], the expected alterations under conditions of energy deficiency were not observed. Nevertheless, the tendency toward lower AgRP levels in the malnourished group suggests that AgRP may still contribute to the regulation of energy balance in interaction with other neuropeptides and metabolic factors [34,35,36]. Further longitudinal studies are required to clarify the role of AgRP in malnutrition.

In the present study, no statistically significant difference in POMC levels was observed between the groups; however, POMC levels tended to be higher in malnourished children compared with healthy controls. POMC, a precursor peptide synthesized in the hypothalamic arcuate nucleus, plays a central role in energy homeostasis through its anorexigenic effects [37]. Although POMC levels are generally expected to be suppressed under conditions of malnutrition, previous evidence suggests that reactive upregulation of POMC expression may occur during the recovery or treatment phases of chronic malnutrition in parallel with restored energy intake [38]. Furthermore, even in the presence of low post-malnutrition leptin levels, increased leptin sensitivity could reactivate the POMC neurons [39]. In addition, conditions such as infection, systemic inflammation, or psychological stress may stimulate the production of adrenocorticotropic hormone (ACTH), which is another product of POMC, thereby increasing the overall POMC level [40]. Thus, an increased POMC level may also reflect a homeostatic adjustment process following the acute phase of malnutrition. In conclusion, the unexpected elevation in POMC levels may represent a multifaceted response encompassing metabolic, hormonal, and neuroendocrine adaptations.

Peripherally derived gastrointestinal peptides also play an important role in the development of malnutrition [41]. Ghrelin and PYY are two key gut peptides with opposing effects in the regulation of energy homeostasis. Postprandial secretion of PYY suppresses food intake through ghrelin-sensitive hypothalamic neurons [42,43]. In our study, the association between elevated PYY levels and reduced food intake suggests that PYY may contribute to undernutrition. Moreover, the inhibitory effect of PYY on POMC neurons may explain the absence of a significant difference in POMC levels in malnourished patients [44,45].

Childhood malnutrition is a multifactorial condition influenced by inadequate nutrient intake, inappropriate feeding practices, food insecurity, and socioeconomic factors [46]. Although socioeconomic inequality is frequently cited as a major contributor to malnutrition [47,48], our study did not identify a significant association between malnutrition and either socioeconomic status or parental education.

Various tools have been developed to assess eating behaviors in children with no defined organic causes. One such tool, the CEBQ, is primarily designed to evaluate the diversity of eating styles among children [49]. In our study, no significant correlations were found between the two groups in terms of the subscales, such as food responsiveness, emotional overeating, enjoyment of food, desire to drink, satiety responsiveness, slowness in eating, emotional undereating, and food fussiness. Consequently, we concluded that the variability in eating styles among the children did not appear to influence malnutrition. Since the CEBQ has previously been used predominantly in overweight and obese pediatric populations, the inclusion of malnourished children in this study enhances its originality and scientific value [50].

Contrary to the literature, no significant association was found between body composition—which is typically expected to be impaired due to nutritional deficiency—and sleep quality or duration in this study. However, the mean sleep duration in the malnourished group was markedly below age-appropriate recommendations. According to the National Sleep Foundation, the minimum recommended sleep duration is 9–11 h for school-aged children and 8–10 h for adolescents [51], whereas the mean sleep duration in our malnourished cohort was 6.85 ± 1.32 h. Previous findings indicating that severe sleep restriction can reduce appetite and energy intake [52,53] suggest that sleep duration may be an important factor to consider in the context of energy balance in malnourished children.

Laboratory findings contributed to the evaluation of malnutrition in this study. Although serum albumin is known to be influenced by inflammatory processes and may not always reliably reflect nutritional status [54,55], the significantly lower albumin levels observed in malnourished children without chronic disease suggest that it may still provide supportive information regarding nutritional deficiency in pediatric populations [52]. In addition, micronutrients play a critical role in growth and development, and our study demonstrated a significant association between serum vitamin D levels and both stunting and underweight status; however, the relationship between vitamin D deficiency and malnutrition may vary depending on population-specific and methodological differences [53,54,56].

A major strength of the present study is its comprehensive approach, which incorporates a wide range of confounding factors, including age, parental education level, socioeconomic status, anthropometric measurements, the CSHQ, and the CEBQ. To the best of our knowledge, this is the first study to concurrently investigate sleep habits, eating behaviors, and circulating levels of orexin A, AgRP, POMC, and PYY in malnourished children.

## 5. Limitations

This study has several limitations. During the study process, limited cooperation from children and their parents was observed. Data were collected using self-report questionnaires, which may have led to inattentive responses to certain items. Diurnal variations in peptide levels could not be assessed, nor could repeated measurements be performed to evaluate associations with eating and sleep questionnaires. In addition, polysomnography could not be performed due to practical, ethical, and logistical constraints in the pediatric population.

Although plasma levels of orexin A, AgRP, POMC, and PYY were measured to assess appetite-related peptide activity, their concentrations in cerebrospinal fluid (CSF) were not evaluated. This represents an important limitation, as CSF sampling is ethically restricted in pediatric populations due to its invasive nature and is technically challenging because of age-related physiological variability and procedural difficulties. Therefore, assessment of CSF peptide levels in pediatric studies is often not feasible due to biological and methodological constraints [57].

Due to the cross-sectional design of this study, the relationships between appetite-regulating neuropeptides and malnutrition do not allow causal inferences, and the findings should therefore be interpreted with caution. In addition, the study included children with moderate to severe malnutrition and healthy controls, while overweight and obese individuals were excluded. The inclusion of both acute and chronic malnutrition cases may have limited the ability to detect distinct alterations in sleep and eating behaviors, particularly in children with acute malnutrition. Finally, considering age-related differences in sleep duration, evaluating participants in separate age groups (children and adolescents) might have yielded more age-specific insights.

## 6. Conclusions

The findings of this study revealed that plasma levels of orexin A and PYY were significantly higher in the malnourished children compared to the healthy controls, independent of age, sex, and BMI. Notably, a positive correlation was identified between orexin A levels and emotional undereating behavior, which suggests that this neuropeptide may play a role not only in energy homeostasis but also in the emotional components of eating behavior. The data suggest that orexin A and PYY may be involved in the etiopathogenesis of malnutrition. Due to their appetite-regulating potential, these peptides may serve as clinically meaningful, noninvasive biomarkers in malnourished pediatric patients. However, the relationship of appetite- and sleep-regulating peptides with malnutrition, their capacity to detect changes in eating behavior, and their clinical utility remain to be fully elucidated. Therefore, further research with larger sample sizes and longitudinal study designs is necessary.

## Figures and Tables

**Table 1:** Demographic and anthropometric characteristics of the study groups.

	Malnourished Children (99)	TD (85)	t/x^2^/z	*p*	D
Age	9.61 ± 2.99	10.16 ± 3.70	−1.110	0.269	2.552
Sex	59 girls 40 boys	54 girls 31 boys	0.299	0.585	0.040 ^a^
Socioeconomic Status	$500 (35) $500–$1000 (51) $1000–$2000 (13)	$500 (30) $500–$1000 (44) $1000–$2000 (11)	0.002	0.999	0.003 ^a^
Age of mother	36.37 ± 6.01	36.30 ± 6.45	0.074	0.941	0.001
Mother’s highest level of education	Primary School (4) Secondary School (33) High School (36) University (16) Master’s/PhD (9)	Primary School (4) Secondary School (22) High School (35) University (15) Master’s/PhD (9)	2.730	0.842	0.122 ^a^
Age of the father	39.67 ± 6.03	39.52 ± 6.41	0.160	0.873	0.002
Father’s highest level of education	Primary School (5) Secondary School (29) High School (24) University (31) Master’s/PhD (10)	Primary School (4) Secondary School (22) High School (23) University (26) Master’s/PhD (10)	0.501	0.992	0.052 ^a^
Weight	22.76 ± 8.04	26.28 ± 12.97	−2.240	0.026	0.061
Weight-p ^b^	1.54 ± 1.46	44.28 ± 28.81	−11.560	<0.001	1.260
Weight SDS ^b^	−2.36 ± 0.59	0.62 ± 6.90	−11.615	<0.001	0.355
Height ^b^	124.55 ± 20.37	126.40 ± 27.13	−0.435	0.664	0.518
Height-p ^b^	7.52 ± 8.40	47.13 ± 28.35	−10.197	<0.001	0.448
Height SDS ^b^	−1.64 ± 0.65	0.02 ± 1.15	−10.037	<0.001	1.731
BMI ^b^	13.57 ± 1.53	17.04 ± 3.69	−8.343	<0.001	0.122
BMI P ^b^	4.92 ± 7.91	41.48 ± 30.51	−9.742	<0.001	0.129

TD = Typically Developing; D = Cohen’s d effect size; SDS = Standard Deviation Score; *p* = Percentile; BMI = Body Mass Index; t/x^2^/z = Test statistics (*t*-test, chi-square, z-score); $ = dollar. ^a^ Cramer’s V effect size. ^b^ Mann–Whitney U test.

**Table 2:** Comparison of sleep and eating behavior scale scores between malnourished children and the control group.

	Malnourished Children (99)	TD (85)	t/x^2^/z	*p*	D
Bedtime resistance	11.03 ± 1.98	11.11 ± 1.98	−0.297	0.766	0.004
Sleep duration ^b^	6.85 ± 1.32	6.68 ± 1.25	−0.723	0.470	0.136
Sleep anxiety ^b^	6.02 ± 2.11	6.25 ± 2.05	−0.972	0.331	0.114
Night wakings	4.21 ± 1.23	4.31 ± 1.22	−0.581	0.562	0.085
Parasomnias ^b^	9.05 ± 2.37	9.37 ± 2.55	−0.991	0.322	0.132
Sleep-disordered breathing ^b^	3.47 ± 0.92	3.47 ± 0.86	−0.215	0.830	0.004
Sleepiness during the day	14.25 ± 2.50	14.50 ± 2.67	−0.664	0.508	0.009
CSHQ	54.41 ± 6.60	55.24 ± 6.97	−0.831	0.407	0.012
Food Enthusiast	9.60 ± 4.56	10.07 ± 4.33	−0.705	0.482	1.933
Emotional Overeating (CSHQ) ^b^	7.11 ± 3.49	7.88 ± 3.95	−1.445	0.148	0.206
Enjoying food (CSHQ)	13.30 ± 4.33	13.94 ± 4.30	−0.999	0.319	0.014
Desire to Drink (CSHQ)	8.49 ± 3.05	8.80 ± 2.98	−0.682	0.496	2.809
Satiety Responsiveness (CSHQ)	22.93 ± 5.14	22.05 ± 5.29	1.142	0.255	0.016
Slowness in Eating(CSHQ)	10.66 ± 3.75	10.82 ± 3.50	−0.291	0.771	0.004
Emotional undereating (CSHQ)	11.75 ± 3.41	11.42 ± 3.30	0.672	0.503	0.009
Food Fussiness (CSHQ) ^b^	8.18 ± 4.91	8.16 ± 4.16	−0.308	0.758	0.003

TD = Typically Developing; D = Cohen’s d effect size; CSHQ = Children’s Sleep Habits Questionnaire (CSHQ) total score; Food Fussiness = Children’s Eating Behaviour Questionnaire; t/x^2^/z = Test statistics (*t*-test, chi-square, z-score); ^b^ Mann–Whitney U test.

**Table 3:** Comparison of blood levels between the two groups.

	Malnourished Children (99)	TD (85)	t/x^2^/z	*p*	D
Hb	12.92 ± 1.10	13.27 ± 1.39	−1.890	0.060	0.027
Albumin	4.29 ± 0.41	4.64 ± 0.29	−6.453	<0.001	0.965
Vitamin D	15.48 ± 7.96	25.55 ± 10.61	−7.336	<0.001	0.177
Ferritin ^b^	37.31 ± 21.39	43.48 ± 21.87	−2.482	0.013	0.285
Vitamin B12	413.16 ± 147.22	388.71 ± 122.25	1.213	0.227	0.180
Folic acid	8.25 ± 3.62	8.74 ± 3.02	−0.999	0.319	0.148
Orexin A ^b^	70.67 ± 135.79	41.66 ± 42.80	−3.518	<0.001	0.913
AgRP ^b^	20.38 ± 29.49	23.46 ± 26.49	−1.877	0.061	0.110
POMC ^b^	0.44 ± 0.57	0.36 ± 0.11	−0.809	0.419	0.188
PYY ^b^	106.86 ± 81.94	84.33 ± 8.22	−7.051	<0.001	0.897

TD = Typically Developing; D = Cohen’s d effect size; AgRP = Agouti-related peptide; POMC = Pro-opiomelanocortin; PYY = Peptide YY; Hb = Hemoglobin; t/x^2^/z = Test statistics (*t*-test, chi-square, z-score); ^b^ Mann–Whitney U test.

**Table 4:** Correlation Between Appetite-Regulating Peptides and Sleep Parameters from the Children’s Sleep Habits Questionnaire (CSHQ).

		Bedtime Resistance	Sleep Duration	Sleep Anxiety	Night Wakings	Parasomnias	Sleep- Disordered Breathing	Daytime Sleepiness	CSHQ
Orexin A	*p*	0.892	0.889	0.821	0.904	0.547	0.427	0.588	0.080
	*r*	0.014	0.014	−0.023	0.012	0.061	0.081	0.055	0.177
AgRP	*p*	0.866	0.994	0.437	0.544	0.704	0.169	0.127	0.646
	*r*	−0.017	0.001	−0.079	−0.062	−0.039	−0.139	−0.154	−0.047
POMC	*p*	0.598	0.673	0.955	0.066	0.334	0.634	0.876	0.971
	*r*	−0.054	0.043	0.006	0.186	−0.098	−0.048	0.016	−0.004
PYY	*p*	0.814	0.690	0.856	0.381	0.568	0.840	0.531	0.474
	*r*	−0.024	0.041	−0.018	−0.089	−0.058	−0.021	−0.064	−0.073

AgRP: agouti-related protein, POMC: proopiomelanocortin, PYY: peptide YY. *p*: Probability value, *r*: Correlation coefficient.

**Table 5:** Correlation Between Appetite-Regulating Peptides and Subscales of the Children’s Eating Behaviour Questionnaire (CEBQ).

		Food Responsiveness	Emotional Overeating	Enjoyment of Food	Desire to Drink	Satiety Responsiveness	Slowness in Eating	Emotional Undereating	Food Fussiness
Orexin A	*p*	0.638	0.212	0.704	0.398	0.612	0.037	0.239	0.515
	*r*	0.048	0.127	0.039	−0.086	−0.052	0.210	−0.119	0.066
AgRP	*p*	0.683	0.602	0.530	0.308	0.263	0.367	0.623	0.815
	*r*	0.042	−0.053	−0.064	0.103	0.114	0.092	−0.050	−0.024
POMC	*p*	0.922	0.337	0.537	0.921	0.476	0.359	0.980	0.378
	*r*	0.010	−0.098	−0.063	−0.010	0.072	0.093	−0.003	−0.090
PYY	*p*	0.691	0.689	0.267	0.266	0.169	0.058	0.966	0.758
	*r*	−0.040	−0.041	−0.113	0.113	0.139	0.191	−0.004	0.031

AgRP: agouti-related protein, POMC: proopiomelanocortin, PYY: peptide YY. *p*: Probability value, *r*: Correlation coefficient.

**Table 6:** Hierarchical regression results.

Stage 1, Regression Results	Coefficient (β)	SE	Wald Statistics	*p*	95% CI
Constant	8.762	1.696	26.692	<0.001	5.438, 12.087
Orexin A	0.004	0.004	1.050	0.286	−0.003, 0.012
AgRP	−0.003	0.007	0.196	0.645	−0.016, 0.01
**Stage 2, Regression Results**	**Coefficient (β)**	**SE**	**Wald Statistics**	***p***	**95% CI**
Constant	−1.941	2.553	0.578	0.447	−6.945, 3.062
POMC	−6.366	2.604	5.977	0.014	−11.47, −1.263
PYY	0.154	0.033	21.976	<0.001	0.089, 0.22
**Stage 3, Regression Results**	**Coefficient (β)**	**SE**	**Wald Statistics**	***p***	**95% CI**
Constant	−3.411	2.842	1.440	0.23	−8.981, 2.158
Orexin A	0.007	0.006	1.400	0.226	−0.004, 0.019
AgRP	−0.007	0.007	1.178	0.289	−0.022, 0.006
POMC	−6.249	2.577	5.881	0.015	−11.301, −1.198
PYY	0.166	0.036	21.262	<0.001	0.096, 0.236

AgRP: agouti-related peptide, POMC: proopiomelanocortin, PYY: peptide YY, SE: standard error.

## Data Availability

The data presented in this study are available on request from the corresponding author. The data are not publicly available due to ethical and privacy restrictions related to human participants.

## Abbreviations

The following abbreviations are used in this manuscript:

AgRP	Agouti-related protein
APC	Article Processing Charge
ARC	Arcuate nucleus
BMI	Body Mass Index
CEBQ	Children’s Eating Behaviour Questionnaire
CI	Confidence Interval
CRediT	Contributor Roles Taxonomy
CSF	Cerebrospinal fluid
CSHQ	Children’s Sleep Habits Questionnaire
ELISA	Enzyme-Linked Immunosorbent Assay
GABA	Gamma-Aminobutyric Acid
Hb/Hgb	Hemoglobin
HPA axis	Hypothalamic–Pituitary–Adrenal axis
LLR	Log-Likelihood Ratio
MC3R/MC4R	Melanocortin receptor ¾
MSH	Melanocyte-Stimulating Hormone
NPY	Neuropeptide Y
POMC	Proopiomelanocortin
PYY	Peptide YY
R^2^	(Pseudo) R-squared
REM	Rapid Eye Movement
SDS	Standard Deviation Score
SE	Standard Error
SPSS	Statistical Package for the Social Sciences
TD	Typically Developing
WHO	World Health Organization
